# Hypocoagulation induced by broad-spectrum antibiotics in extensive burn patients

**DOI:** 10.1186/s41038-019-0150-7

**Published:** 2019-04-26

**Authors:** Jian Liu, Yiqing Liu, Shengjun Liu, Qin Zhang, Jiexin Zheng, Yiwen Niu, Xuefeng Wang

**Affiliations:** 1 Department of Burn & Plastic Surgery, Ruijin Hospital, Shanghai Jiaotong University, School of Medicine, Shanghai, China; 2 Clinical Medicine, Shanghai Jiaotong University, School of Medicine, Shanghai, China; 3 Transfusion Department, Ruijin Hospital, Shanghai Jiaotong University, School of Medicine, Shanghai, China

**Keywords:** Hypocoagulation, Extensive burns, Vitamin K deficiency, Antibiotic-induced coagulopathy

## Abstract

**Background:**

Patients with extensive burns usually develop pro-coagulation soon after the injury if there is no sepsis occurred. We describe the case of an extensive burn adult suffering from hypocoagulation not related to sepsis, but secondary to antibiotic treatment.

**Case presentation:**

Here, we report a case of an adult male patient suffering from flame burns of 45% total body surface area (40% full thickness) combined with inhalation injury. Hypocoagulopathy with soaring prolonged activated partial thromboplastin time value occurred on third week post-burn while systemic infection had been under control by application of broad-spectrum antibiotics. Investigations showed that not the infection but vitamin K-related coagulation factor deficiency were responsible for unexpected bleeding. However, supplemental vitamin K was not the key as we expected, which prompted us trying to decode the underlying cause of coagulation disturbance in this patient and pick out the most effective treatment for live-saving. After the withdrawal of highly suspected broad-spectrum antibiotic, Meropenem®, disturbed vitamin K related coagulation factors gradually restored to their optimal levels so as to maintain normal coagulation status. Therefore, surgical procedures without further risk of bleeding could be carried out in time for wound recovery. The patient was discharged on post-burn day 67 and transferred to a secondary hospital for his rehabilitation.

**Conclusion:**

Hypocoagulopathy may be devoted to different reasons other than sepsis in extensive burns. Early recognition of the cause for coagulation disturbance is critical to make appropriate treatment and save patients’ lives. This case illustrated the importance of unveiling the mist cause for coagulation disturbance occurred in extensive burn patient, which paved the way for optimal life-saving treatments. And we also recommend burn surgeons to be alerted to antibiotic-induced vitamin K deficiency-related coagulopathy among critical burn patients.

## Background

Patients with extensive burns usually develop pro-coagulation soon after the injury if there is no sepsis occurred. Hypocoagulation seldom comes upon critically burned patients, except in those with severe infection or sepsis with dissemination intravascular coagulation (DIC). We describe the case of an extensive burn adult suffering from hypocoagulation not related to sepsis, but secondary to antibiotic treatment.

## Case presentation

**Fig. 1:**
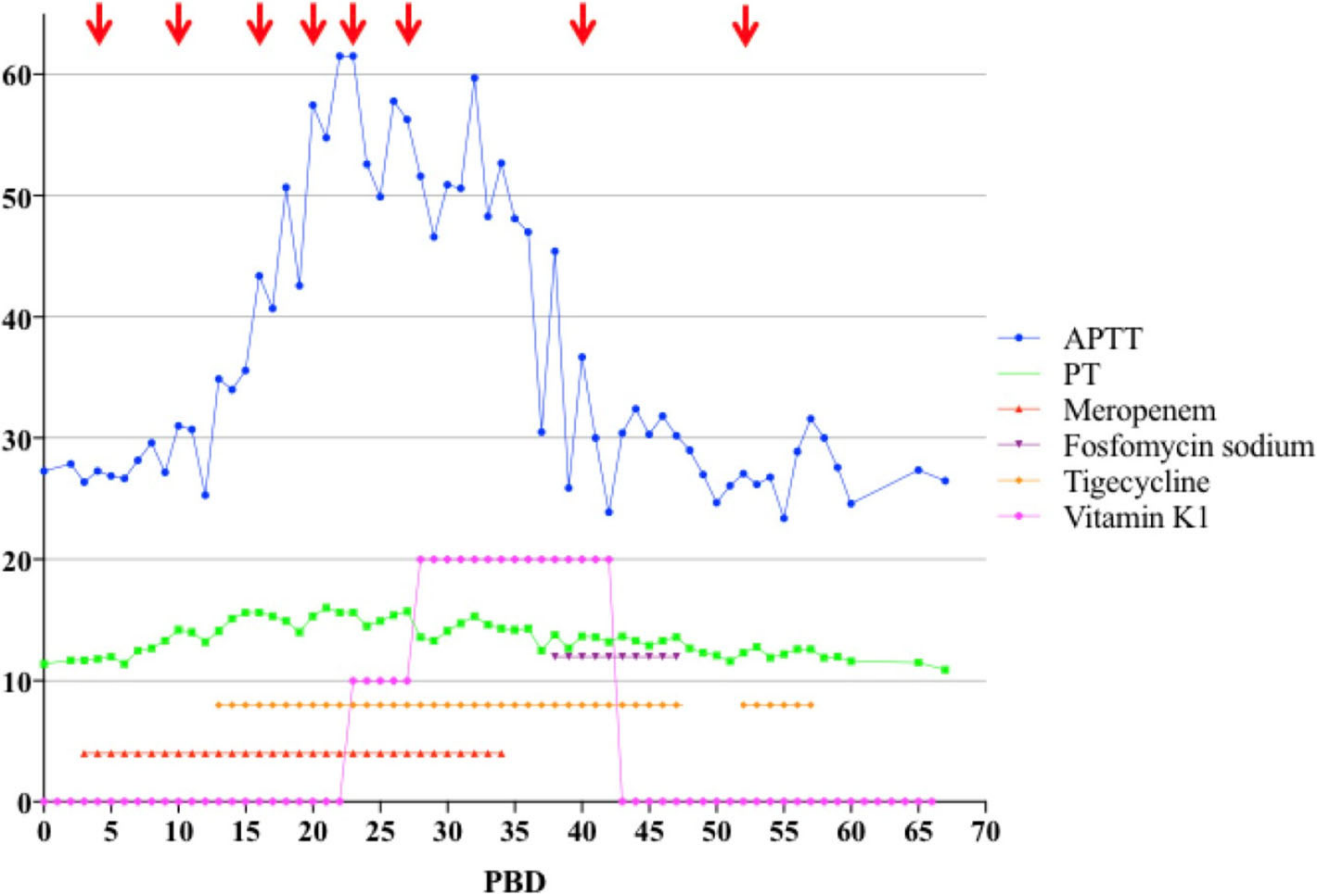
Chronological relationships of antibiotic treatment, vitamin K1 administration, surgical procedures, and coagulation time fluctuation of the patient. *APTT* prolonged activated partial thromboplastin time, *PBD* post-burn day, *PT* prothrombin time

**Fig. 2:**
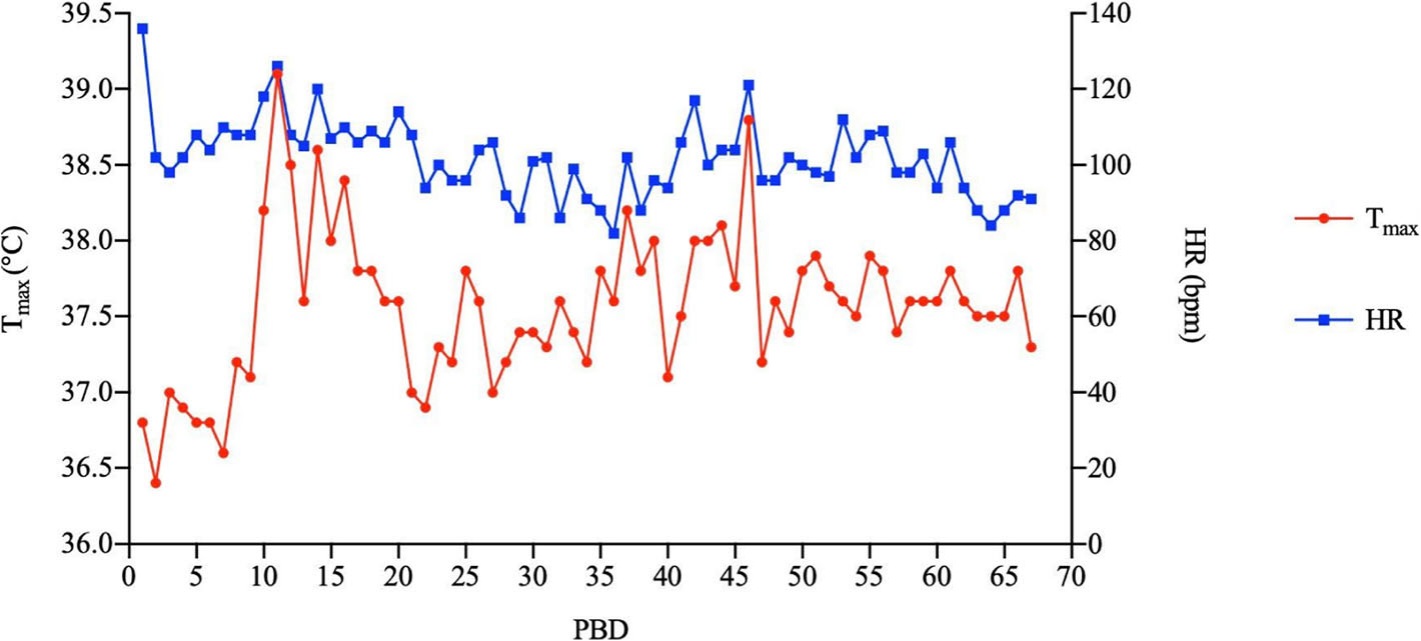
Daily maximal temperature and heart rate (HR) of the patient during his stay in hospital. *PBD* Post-burn day

**Fig. 3:**
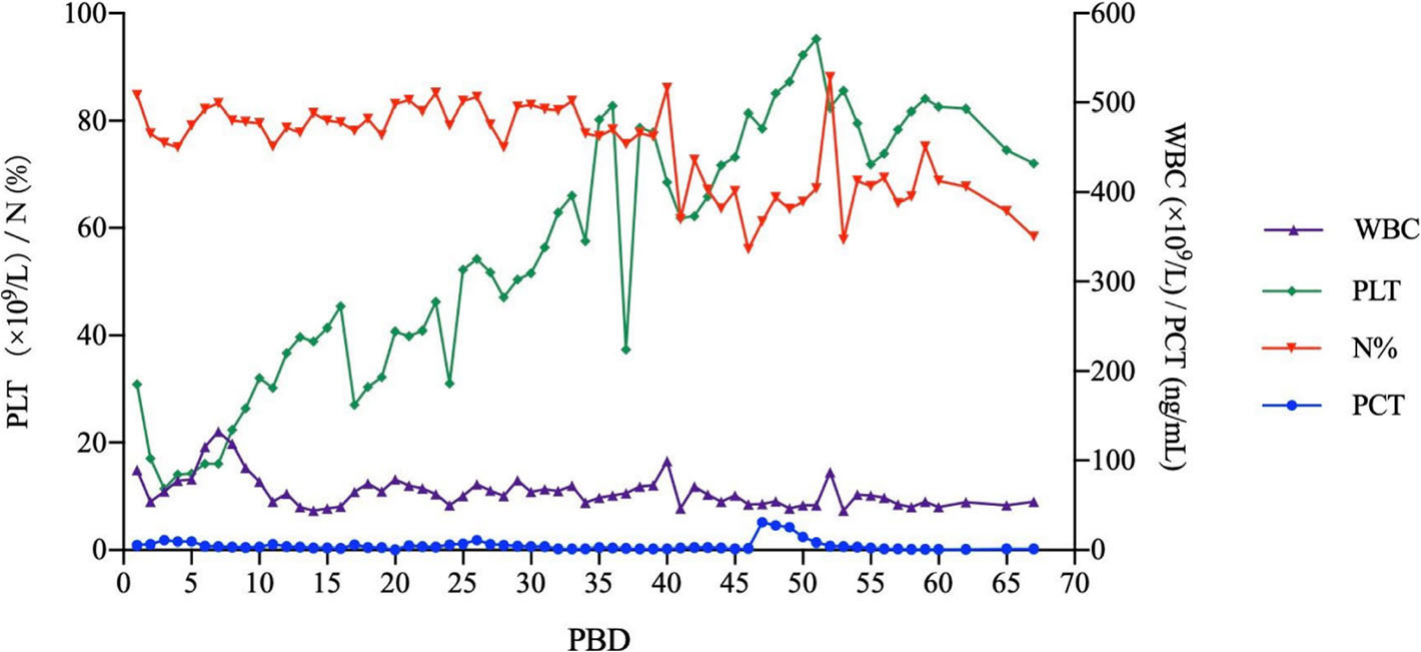
Infection related indicators (blood cell count and Precalcitonin (PCT)) levels of the patient during his stay in hospital. *PBD* Post-burn day, *PLT* Platelet, *WBC* White blood cell

A 40-year-old male, weighing 65 kg, suffered from flame burns of 45% total body surface area (TBSA) (40% full thickness) combined with inhalation injury, affecting his face, neck, trunk, upper extremities, and right lower extremity. He was rescued by the firemen from the accidental site and directly sent to the local hospital, which provided sufficient fluid resuscitation during hypovolemic stage post-burn. Negative personal medical history and family history were confirmed while the patient was transferred to our hospital for further treatment on the post-burn day (PBD) 3. Topical usage of 1% silver sulfadiazine (1%SD-Ag cream) was served as wound management together with surgical procedures of escharectomy (upper extremities and right lower extremity) and heterograft on PBD 4, consecutively 2 autografting on PBD 10 and 16. Broad-spectrum antibiotics, Meropenem® (1.0 g three times daily), was applied intravenously since PBD 3 as the empirical therapy for extensive burn patients and continued for ensured *Pseudomonas aeruginosa*-positive of the wound in this patient (Fig. 1). Calorie intake of Fresubin® 1000 mL/day was commenced on PBD 5 via gastrointestinal (GI) tube as a supplemental nutrition other than oral intake of normal food, except on those days of surgical procedures. Not until PBD 13 did the patient show the signs of infection, such as chill, high fever, wounds infiltration, delirium, and neutrocytosis (Figs. 2 and 3), while his hemodynamic status remained stable, all coagulation criteria within normal range. Wound and blood culture were both reported *Klebsiella pneumoniae*-positive (multi-drug-resistant strain, tigecycline medium). Hence, treatment with tigecycline (0.5 g three times daily) was initiated intravenously on PBD 13, together with Meropenem®, and worked well in controlling those infections till PBD 18. All lab findings coordinate with the severity of the patient during that period of time, including his coagulation status.

Nevertheless, concurrent with remission of signs of severe infection, there came the abnormal oozing of blood on the donor site during the third autografting and uncontrolled bleeding while removing the central venous line on PBD 23, which related to the prolonged activated partial thromboplastin time (APTT) 61.5 s, normal range 25.1–39.5 s, while prothrombin time (PT) 15.6 s, normal range 10.0–16.0 s, and the international normalized ratio (INR) 1.32 were normal. An intravenous bolus of 10 mg vitamin K1 was applied during the procedure and continued in the following 5 days. Further investigation of the patient’s coagulation status was launched after the operation. There was no sign of DIC at this time, with normal value of platelets (227 × 10^12^/L), fibrin degradation products (FDP) 4.7 mg/L, normal range 0-5 mg/L, D-dimer 1.68 mg/L, normal range < 0.55 mg/L, and slightly reduced fibrinogen (Fg) 1.8 g/L, normal range 1.8–3.5 g/L. Complete screening of patient’s coagulation factors was carried out on PBD 26 (Table 1). von Willebrand factor (vWF) level and activity were within the normal range, lupus anticoagulant (LAC) was negative, and coagulation factor VIII and V were normal, deficiency of multiple coagulation factors (Table 1: coagulation factor II, coagulation factor VII, coagulation factor IX, and coagulation factor X activities were 39%, 35%, 45.1%, and 28% respectively on PBD 26) that related to vitamin K deficiency (VKD) was indicated as the reason for coagulopathy in this patient. Unfortunately, specific detection of serum vitamin K concentration and protein induced in vitamin K deficiency (PIVKD) were not available in our hospital. However, what really mattered was to relief further risk of bleeding and coagulation crash as quickly as possible. Thereafter, supportive therapies, such as 200 ml fresh frozen plasma (FFP), was administered daily for consecutive days and 600 IU prothrombin complex was infused on PBD 26 when the patient was diagnosed of hypothrombinemia. Although the coagulation status remained abnormal on PBD 27, surgical debridement of the neck, trunk, and right lower extremity and grafting were performed. There was no abnormal bleeding either on the donor site or burn wounds, and wound healing of any sites was not interfered. At the same time, application of vitamin K1 intravenously increased to 20 mg daily for another 15 days, but showed mild effect to reverse the surging APTT level.

**Table 1:** Coagulation factors values on post-burn day (PBD) 26

Activity	Factor								
	vWF	LAC	VIII	IX	X	VII	II	XI	XII
Normal range	60–150%	< 1.2	50–150%	50–150%	50–150%	50–150%	50–150%	50–150%	50–150%
PBD 26	219.2%	1.01	151.1%	45.1%	28%	35%	39%	53.2%	58.9%

*vWF* von Willebrand Factor, *LAC* Lupus Anticoagulant

**Fig. 4:**
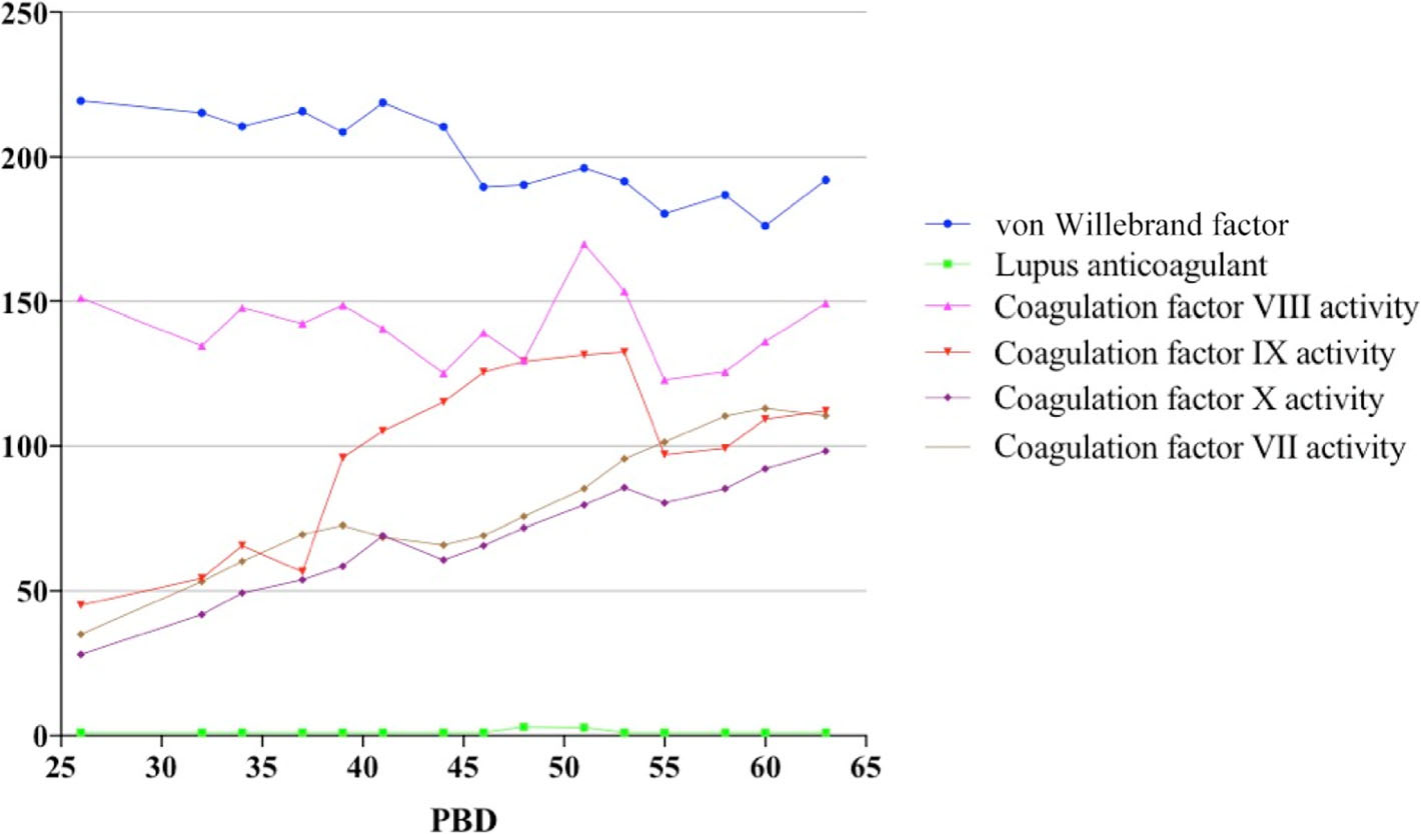
Coagulation factor level fluctuation of the patient before and during his treatment of hypocoagulation. *PBD* Post-burn day

**Fig. 5:**
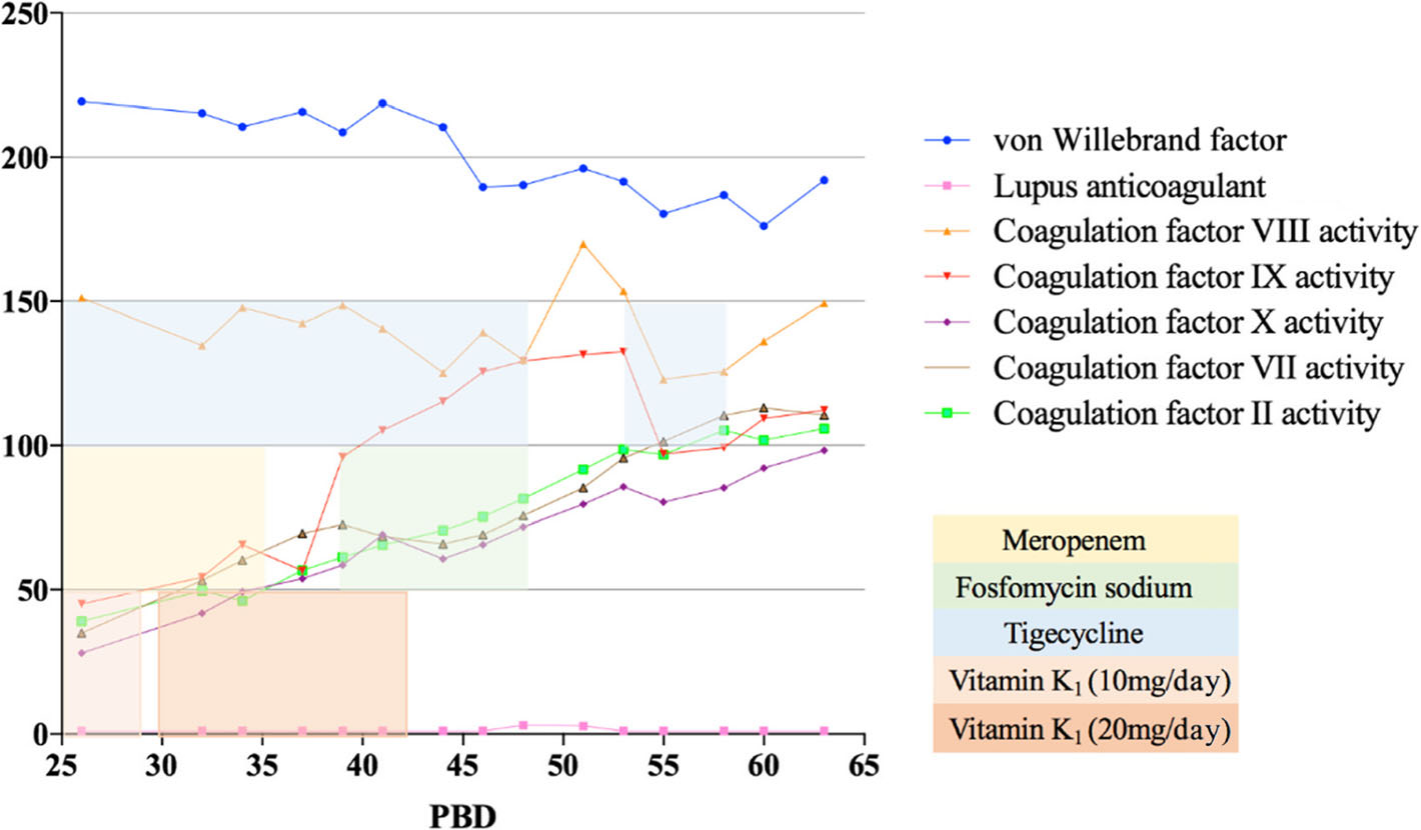
Relationship between intravenous vitamin K1, meropenem, fosfomysin sodium, tigecycline, and levels of factors related to hypocoagulopathy. Meropenem, 1.0 g three times daily; tigecycline, 0.5 g three times daily; fosfomycin sodium, 4.0 g three times daily. *PBD* post-burn day

Nothing should be considered as the first priority, except finding out the exact reason for VKD. Concerning no short of vitamin K intake, no impairment of liver function, and vitamin K recycling occurred in this case, but scarcity of endogenous vitamin K generated by intestinal bacterial flora soon came to our view. Meropenem® application was discontinued on PDB 34. Interestingly, 2 days after, APTT improved to 47 s and came back to the normal range on PDB 39 along with normal value of coagulation factor II, VII, IX, and X (Figs. 1 and 4), even with combined usage of tigecycline (0.5 g three times daily) and fosfomycin sodium (4.0 g three times daily) intravenously, started on PBD 38, dealing with multi-resistant strain of *Klebsiella pneumonia* (tigecycline resistant). Another two operations were performed for grafting on PBD 40 and 52. Tigecycline and fosfomycin sodium were applied in the same dosage as previously used for multi-drug-resistant *Klebsiella pneumonia* still detected in wound culture. The patient was discharged on PBD 67 with all burn wounds recovered and no abnormality in coagulation status (Fig. 5).

## Discussion

Patients with severe burns usually develop pro-coagulation soon after the injury. Elevated fibrinogen levels are often present immediately following burn injury, owing to the shift in hepatic protein synthesis to acute-phase proteins including complements, interleukins, fibrinogens, and etc. [1], while dilution effects might explain the temporal reduction in the levels of coagulating proteins during the early resuscitation phase with no coagulation function affected. For those combined with inhalation injury, studies confirmed that hypercoagulability occurred among patients after PBD 4 [2, 3]. Furthermore, extensive endothelial impairment ignites coagulation cascade, which intimately intertwined with inflammation reaction following the thermal injury and during infection [4]. And along with the wound recovery, coagulopathy is less related to burn injury but more likely owe to sepsis [5]. In our case, patients’ coagulation status was kept normal at the time of transferring to our burn center and the following 2 weeks, even if the patient had been suffering from severe wound and bloodstream infection since PBD 13. There were no signs of DIC, and coagulation criteria were all within normal range (PT 14.1 s, APTT 34.9 s, fibrinogen was 4.9 ng/L and normal fibrinogen degraded product level). When the first coagulation abnormalities developed on PBD 23, signs and symptoms of severe infection had already been remitted (relief of high fever, delirium, and wound infiltration, also blood culture was reported negative on PBD 24), attributed to wound debridement accompanied with effective systemic antibiotic treatment, and white blood cell counts were elevated within accepted level (10.38 × 10^9^/L, neutrophils 85.1%). Platelet (PLT) counts (277 × 10^12^/L) and precalcitonin (PCT) (0.5 ng/mL) were normal, so the possibility of thrombocytopenia and septic coagulopathy was ruled out. There was no over-transfusion or enormous blood loss during surgical procedures, and serum calcium level was occasionally slightly decreased (fluctuated from 1.82 to 2.05 mmol/L), which indicated no relationship between surgical procedure, blood loss, transfusion, and coagulopathy.

On the other hand, acquired coagulation factor deficiency might be associated with systemic amyloidosis, respiratory mycoplasma infection, and is extremely rare among extensive burn patients but sporadically reported in the literature [6–9]. It could due to the presence of an inhibitor, anti-cardiolipin antibody, or be related to antibiotic treatment, such as Meropenem®, of which the mechanism still remained unknown. In the present case, the patient had a negative medical or family history. The plasma-mixing test, with resultant normalization of PT and APTT contradicted the presence of antibody or inhibitor. Furthermore, not just factor X activity was at an extremely low level (28%, normal range 50 × 150%), but other coagulation factors related to vitamin K metabolism (coagulation factor II, coagulation factor VII, coagulation factor IX, and coagulation factor X activities were 39%, 35%, and 45.1% respectively) were all impaired significantly.

Vitamin K acts as the cofactor for γ-glutamyl carboxylase that is responsible for the post-translational modification of glutamate residues in of coagulation factor II, VII, IX, and X [10]. VKD may reduce biological activity of these factors presenting with increased PT or APTT and clinically manifested with bleeding. Serum concentration of phylloquinone (vitamin K1), vitamin K1 2,3-Epoxide (K1O), and protein induced by vitamin K absence or antagonist II (PIVKA-II) can be measured; however, the diagnosis is usually clinical and by exclusion [11]. Dietary insufficiency and long-term broad-spectrum antibiotic therapy could be the reason for VKD among critically ill patients. For our patient, the first possibility was easy to be ruled out since normal diet and supplemental GI nutrition out-reached 65 μg/day, the recommended nutrient intake of vitamin K by the World Health Organization for adult males [12] or the daily requirement of 100 mg phylloquinone recommended for the maintenance of hemostasis [13]. Vitamin K can be obtained not only from digestive food, but also synthesized by intestinal bacterial flora colonizing in the colon or the distal ileum (vitamin K2, menaquinone-MK), that is activated in the liver [14]. In our opinion, patients’ hepatic function tests kept normal when he was hospitalized. No cholestasis or hepatic pathological entities found, which rejected the hypothesis of vitamin K recycle interdiction. Therefore, came the high suspicion of broad-spectrum antibiotics as the reason for secondary VKD that led to hypocoagulopathy in this case.

Antibiotic-induced secondary VKD can develop by the following possible mechanisms: the first is the inhibition of vitamin K metabolism in the liver. Cephalosporins, which include the N-methylthiotetrazole (NMTT) side chain (e.g., cefmetazole, cefotetan, cefamandole, cefoperazone, and moxalactam) or the 2-methyl− 1,3,4-thiadiazole-5-thiol side chain (e.g., cefazolin), may inhibit vitamin K epoxide reductase in the vitamin K metabolism cycles and makes it accumulated in the liver. Usually, patients on parenteral nutrition are at stake of the risk [15]. Low plasma vitamin K1 concentration can ensure the co-relationship between antibiotic application and secondary VKD. It was reported that the average time from the initiation of antibiotic therapy to diagnosis of hypoprothrombinemia was 5.7 days (range, 2–15 days), and patients’ prognosis was well after being administered vitamin K supplements [16]. Obviously, this was not the key in our case since no cephalosporins had been applied. The second mechanism of antibiotic-induced VKD is that broad-spectrum antibiotics suppress intestinal flora that synthesize vitamin K2 (menaquinone-MK), which might play the causative role in our case leading to the clinical manifestation of bleeding. Meropenem® is the bactericide of which the anti-bacterial spectrum covers Gram-positive, Gram-negative bacteria (including *Pseudomonas*), and anaerobic bacteria. There was the report that Meropenem® induced the transient coagulation factor X deficiency of a 12-month-old boy suffering from 25% TBSA and underwent hypocoagulability 3 days after the initiation of the antibiotic. And it is also considered to be responsible for drug-induced immune thrombocytopenia (DITP) that shows acute thrombocytopenia in patients [17]. The mechanism of Meropenem® to initiate different ways of coagulopathy still needs further investigation; however, in present case treatment, long-term usage of Meropenem® might eradicate vitamin K producing intestinal microflora, interdicted the vitamin K2 pathway and made it deprived of vitamin K storage in the patient. Moreover, the combined application of tigecycline might aggravate the clearance of intestinal microflora. Protein induced by vitamin K absence or antagonist II (PIKA-II) is the predictor of VKD in such cases. However, tests investigating specific vitamin K concentrations that might be helpful to the diagnosis were not available in our hospital. Indirect evidences such as recovery of vitamin K-related-coagulation factor values after efficient dosage of supplemental vitamin K bolus (20 mg q.d), clinical and laboratory improvement after discontinuation of Meropenem® gave us the hint that long-term and high dosage of Meropenem® application could be the cause of hypocoagulopathy in this patient.

The role of tigecycline treatment in this case also merits careful consideration. Tigecycline is a tetracycline-class bacteriostatic agent that acts against gram-positive, gram-negative, and anaerobic microbes, but not *Pseudomonas* spp. or *Proteus* spp. [18]. Coagulation disturbances were observed only as an interaction between tigecycline and warfarin. Few, but severe cases of hypofibrinogenemia that related to tigecycline treatment were reported and high dosage of the drug or combination with warfarin could be the risk factors [19, 20]. The mechanism by which tigecycline provokes coagulation disturbance could either relate to the reduction of intestinal microflora or direct on vitamin K activation in the liver. In our case, tigecycline was used to act against the multi-drug-resistant *Klebsiella pneumonia* with recommended dosage, and later combined with fosfomycin sodium when the wound culture proved that the strain shift from medium to resistant to tigecycline. It could have acted synergic with Meropenem® in eradicating intestinal micro-organisms, also according to Naranjo scale [21], which investigates the probability of adverse drug reactions (ADR), indicated that tigecycline could be the doubtful cause for hypocoagulopathy, while Meropenem® might be the possible one. No coagulation disturbance occurred after the re-administration of tigecycline in the case even supplemental vitamin K bolus had been withdrawn at that time.

## Conclusions

Hypocoagulopathy may be devoted to different reasons other than sepsis in extensive burns. Early recognition of the cause for coagulation disturbance is critical to make appropriate treatment and save patients’ lives. Extensive burn patients are vulnerable to secondary VKD related to broad-spectrum antibiotic treatment, but treatable if early supplemental vitamin K is applied. We recommend coagulation parameters be closely monitored and to withdraw antibiotics application as soon as possible. The exact mechanisms of Meropenem® to impair activation of coagulation factors and why there was only endogenous pathway impairment in this case remained to be further elucidated; the potential role of genetic susceptibility could be one way out.

## Abbreviations

ADR: Adverse drug reactions
APTT: Prolonged activated partial thromboplastin time
DIC: Dissemination intravascular coagulation
DITP: Drug-induced immune thrombocytopenia
FDP: Fibrin degradation products
FFP: Fresh frozen plasma
Fg: Fibrinogen
GI: Gastrointestinal
INR: International normalized ratio
K1O: Vitamin K1 2,3-epoxide
LAC: Lupus anticoagulant
NMTT: N-methylthiotetrazole
PBD: Post-burn day
PCT: Precalcitonin
PIVKA-II: Protein induced by vitamin K absence or antagonist II
PIVKD: Protein induced in vitamin K deficiency
PLT: Platelet
PT: Prothrombin time
TBSA: Total body surface area
VKD: Vitamin K deficiency
vWF: von Willebrand factor
